# Gastropleural fistula: Rare entity with unusual etiology

**DOI:** 10.4103/1817-1737.32233

**Published:** 2007

**Authors:** Anshuman Darbari, Shekhar Tandon, G. P. Singh

**Affiliations:** Department C.T.V.S. and K. G. M. University, Lucknow, Uttar Pradesh, India; * *Department of Anaesthesia, K. G. M. University, Lucknow, Uttar Pradesh, India*

**Keywords:** Fistula, gastric, pleura

## Abstract

Gastropleural fistula is a rare condition. We report a case where fistula developed iatrogenically during repeated intercostal drainage tube insertions for empyema.

Gastropleural fistula has been reported after intrathoracic gastric perforation in hiatal hernia, traumatic diaphragmatic hernia with later gastric perforation, perforated malignant gastric ulcer at fundus, extension of subphrenic abscess with gastric perforation, pulmonary resection and gastric bypass operations.[1–5]

Because of the corrosive actions of gastric juice and with nutritional debility associated with this pathology, early diagnosis and prompt surgical treatment of gastropleural fistula are necessary for satisfactory results.

## Case Report

**Figure 1:**
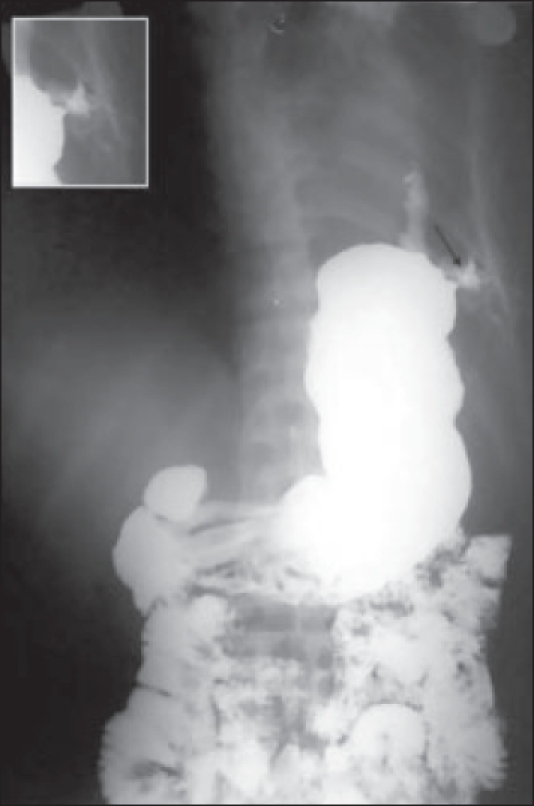
Barium study showing a fistulous tract from fundus of stomach to intercostal tube via left pleural space (marked by arrow). In inset, magnified view of fistula with Malecot's catheter shown

**Figure 2:**
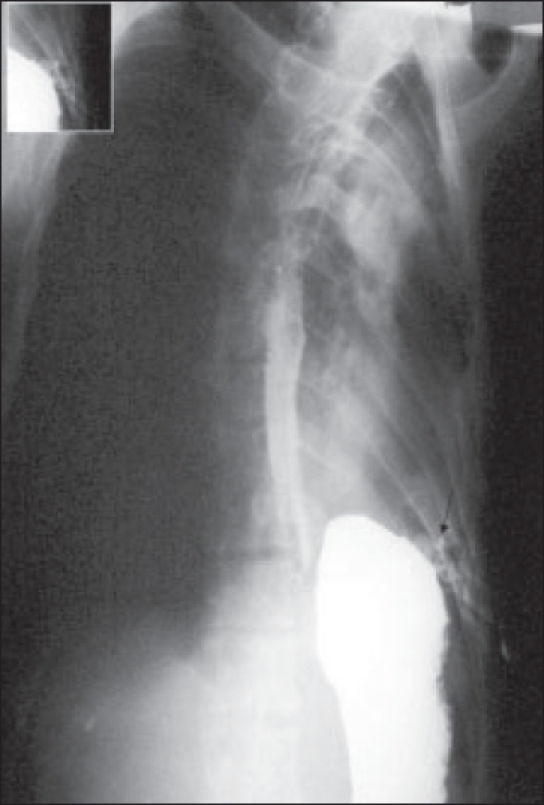
Oblique view of barium study showing normal esophagus and fistulous tract from fundus of stomach to left intercostal tube via pleural space (marked by arrow). In inset, magnified view of fistula with Malecot's catheter shown

A 35-year-old male developed left-sided tubercular empyema thoracis 9 years back. He was maintained on antituberculous drug coverage. A left intercostal tube thoracostomy was done, but no improvement followed. After 3 months, left-sided decortication surgery was done. The lung did not expand completely and he was advised thoracoplasty, which he refused. For residual empyema, intercostal tube was changed repeatedly, nearly once every 6 months. After changing intercostal tube 2 months back, he suddenly noticed the presence of ingesta and food particles in the drainage bottle. He was afebrile but started losing weight. With the suspicion of esophago or gastropleural fistula, barium study was done, which highlighted the opacified pleural cavity with a fistulous tract from fundus of stomach to intercostal tube via left pleural space [Figures 1 and 2]. Examination of pleural fluid revealed odorless, turbid, brownish acidic fluid.

He was advised thoracoplasty and gastropleural fistula closure. At left thoracotomy under general anesthesia, there were dense pleural adhesions; and left pulmonary parenchyma was dystrophic with multiple bronchopleural fistulas. The left hemidiaphragm was raised and after lysis of the adhesions, the gastric fistulous communication of about 2 × 2 cm in left posterolateral part of diaphragm was visualized. The stomach was repaired by continuous monofilament polypropylene suture, and the diaphragm was repaired with continuous monofilament polypropylene suture. Thoracoplasty with resection of four ribs was done. In postoperative phase, minor leak persisted; hence midline laparotomy under general anesthesia was done. Complete separation of gastric fundus from the diaphragm was done, and the fundus rent was repaired by double layer of polygalactin sutures. The diaphragm was repaired with single layer of continuous monofilament polypropylene suture. Feeding jejunostomy was also done. Patient recovered from fistula. Further, rib resection for completion of thoracoplasty is planned.

## Discussion

Markowitz and Herter first described gastropleural fistula in 1960. They described causes of gastropleural fistula as intrathoracic perforation of stomach in hiatal hernia, traumatic diaphragmatic hernia with perforation of stomach and intraperitoneal gastric perforation with erosion of subphrenic abscess via diaphragm.[1,2] Other causes have been subsequently described as complications of pulmonary surgery, esophageal surgery[3,4,7] and gastric bypass operations for morbid obesity. Later it was also recognized that these fistulas might occur in late postoperative phase of esophagogastrectomy, with or without presence of recurrent tumor or radiation therapy.[5–8]

Diaphragm is an effective barrier to spread of infection. Transdiaphragmatic spread of infection occurs by means of spontaneous diaphragmatic perforation. The diagnosis is usually made with contrast radiology, upper GI endoscopy and by testing of pleural fluid. For surgical approaches, both laparotomy and thoracotomy have been described depending on factors as etiology and site of fistula.[3,4,7,8] Previous reports favor laparotomy[3,4,6–8] but we opted for initial thoracotomy, because of infected pleural space with bronchopleural fistula.

Our case is one of this rare diagnosis. Fistula developed likely due to straight and inadvertent forceful insertion of intercostal tube in previous insertion site in seventh left intercostal space. Other significant medical history was decortication surgery for left empyema thoracis 9 years back. To our knowledge, there is no other reported similar case due to this etiology.

This case emphasizes that not only subphrenic infection or below-diaphragmatic pathology can erode and lead to gastropleural fistula, but supradiaphragmatic infection, intrathoracic surgery and strong forceful intercostal tube insertion can also result in this condition.

This case gives us a word of caution for such a rare entity.
